# Supplementary material for: Are the shareholding and trading behaviors of diverse investors affected by the relaxation of day trading?
Source: PLoS One. 2021 Apr 23;16(4):e0250121. doi: 10.1371/journal.pone.0250121 (PMC8064584; doi:10.1371/journal.pone.0250121)
Supplement: Attachment — Submitted filename: Response_to_the_comments_of_reviewers_0219H6.docx [file pone.0250121.s002.docx]

Reviewer #1

1. The theoretical contribution you mentioned in the Introduction is not theoretically justified. There is not much information about what has been researched and what has not. The Introduction is largely occupied with background information which is not really helpful for developing your theoretical arguments. Thus, it is unclear where you position your research in the literature.

**Response** **to valuable comment:**

We really appreciate your treasured comment. We agree with you that we should express clearly what has been researched in this study. Therefore, we generate some supplements to enhance our research theoretically in the literature. The modification is shown in the first three paragraphs of the Introduction from P. 2 to P. 3.

**The following contents are shown in the context of this paper (P. 2-P. 3):**

From the perspective of psychology, there is an environment-behavior theory that discusses the relationship between environmental change and behavior reaction. In theory, the environment is defined as the self-report measures used to assess subjective reactions to that environment and then people evaluate their behavior with regard to the environment accordingly. Besides, the intention is the best predictor of environmental behavior, whereas personal norms, attitudes, and perceived behavioral control have a significant influence on behavioral intention. In sum, the reaction is an attempt to find the results that make changes to the individual as a new thing and to increase knowledge. However, it seems to exist an interesting line of research links psychological influences and financial markets, which evokes our curiosity whether this psychological theory is still functioning for the investment activity. If it does work, we then might establish some investment models for investors to take suitable strategies in advance.

Inspired by the evidence that game rule change might cause behavioral change, we consider that it would be worthwhile to investigate the issue of environment-behavior change in the finance area. We speculate that the regulation change probably results in the change of investor’s behavior in return. On Feb. 1, 2016, Taiwanese government permits to unwind the regulation of day trading in stock market, which makes market participants to believe that the liquidity of market may be improved and the trade of stocks may have more flexibility. Even further, the relaxation may reduce the risk of overnight shareholdings for investors and may protect the interests of investors. However, we argue that there are some issues still unanswered after the relaxation. For example, whether individual and institutional investors, including foreign institutional investors, domestic institutional investors, and security dealers, would prefer trading or holding the stocks with higher day trading ratios, even higher turnover ratios? Does any difference exist for the preference of trading or holding these stocks among individual and institutional investors? These unresolved issues arouse our interest to organize the research.

We conduct this study to examine whether the relaxation of day trading regulation would change the diverse investors' behaviors, which is stemmed from the following motivations. First, we attempt to identify if the speculative individual investors might prefer investing stocks with higher day trading ratios and, in contrast, if the other individual deemed as the uninformative traders might not prefer trading these stocks due to the difficulty of making profits. Second, as realized with making more effort on fundamentals instead of speculation, institutional investors should tend to decrease the shareholdings and trading volume of stocks with higher day trading ratios. However, some institutional investors would prefer trading and holding the stocks with higher day trading ratios, especially for those having unreleased information. Therefore, we endeavor to solve the puzzle of whether these institutional investors have this preference due to liquidity concerns.

2. I hardly see how the three strands of literature are integrated to underpin your conceptual model. There is no clear articulation between the theories and your model and variables.

**Response to valuable comment:**

Thank you for your valuable comment. We agree that we should have more description on the articulation among the three strands of literature and the conceptual model. In this study, we are interested in the reactions of investors’ behaviors after the relaxation of day trading regulation in Taiwan, which is inspired by the environment-behavior theory in psychology. Thus, we conduct the literature review for this study with the diverse investors and day trading as the issues to achieve this goal. Consequently, we add the inspiration and the aim of this study in the first two paragraphs and the third paragraph of the Introduction, respectively. Hopefully, these supplements can link the relations more tightly with the three strands of literature and the conceptual model. The modified text is shown on P. 2 and P. 3.

**The following contents are shown in the context of this paper** **(P. 2 & P. 3):**

From the perspective of psychology, there is an environment-behavior theory that discusses the relationship between environmental change and behavior reaction. In theory, the environment is defined as the self-report measures used to assess subjective reactions to that environment and then people evaluate their behavior with regard to the environment accordingly. Besides, the intention is the best predictor of environmental behavior, whereas personal norms, attitudes, and perceived behavioral control have a significant influence on behavioral intention. In sum, the reaction is an attempt to find the results that make changes to the individual as a new thing and to increase knowledge. However, it seems to exist an interesting line of research links psychological influences and financial markets, which evokes our curiosity whether this psychological theory is still functioning for the investment activity. If it does work, we then might establish some investment models for investors to take suitable strategies in advance.

Inspired by the evidence that game rule change might cause behavioral change, we consider that it would be worthwhile to investigate the issue of environment-behavior change in the finance area. We speculate that the regulation change probably results in the change of investor’s behavior in return. On Feb. 1, 2016, Taiwanese government permits to unwind the regulation of day trading in stock market, which makes market participants to believe that the liquidity of market may be improved and the trade of stocks may have more flexibility. Even further, the relaxation may reduce the risk of overnight shareholdings for investors and may protect the interests of investors. However, we argue that there are some issues still unanswered after the relaxation. For example, whether individual and institutional investors, including foreign institutional investors, domestic institutional investors, and security dealers, would prefer trading or holding the stocks with higher day trading ratios, even higher turnover ratios? Does any difference exist for the preference of trading or holding these stocks among individual and institutional investors? These unresolved issues arouse our interest to organize the research.

We conduct this study to examine whether the relaxation of day trading regulation would change the diverse investors’ behaviors, which is stemmed from the following motivations. First, we attempt to identify if the speculative individual investors might prefer investing stocks with higher day trading ratios and, in contrast, if the other individual deemed as the uninformative traders might not prefer trading these stocks due to the difficulty of making profits. Second, as realized with making more effort on fundamentals instead of speculation, institutional investors should tend to decrease the shareholdings and trading volume of stocks with higher day trading ratios. However, some institutional investors would prefer trading and holding the stocks with higher day trading ratios, especially for those having unreleased information. Therefore, we endeavor to solve the puzzle of whether these institutional investors have this preference due to liquidity concerns.

3. The conceptual development is still weak. Your paper appears to be more like data-driven rather than fundamentally theory-driven. There is no clear Theories and Hypothesis Development" in section 2.

**Response** **to valuable comment:**

We truly appreciate your constructive comments. We agree that we should develop the theory and hypothesis in this study. Therefore, we supplement the theory in the first two paragraphs of the Introduction and the hypothesis in the Literature review section. The revision is presented on P. 2 and P. 6 to P. 9.

**The following contents are shown in the context of this paper** **(P. 2 & P. 6-P. 9):**

**P. 2**

From the perspective of psychology, there is an environment-behavior theory that discusses the relationship between environmental change and behavior reaction. In theory, the environment is defined as the self-report measures used to assess subjective reactions to that environment and then people evaluate their behavior with regard to the environment accordingly. Besides, the intention is the best predictor of environmental behavior, whereas personal norms, attitudes, and perceived behavioral control have a significant influence on behavioral intention. In sum, the reaction is an attempt to find the results that make changes to the individual as a new thing and to increase knowledge. However, it seems to exist an interesting line of research links psychological influences and financial markets, which evokes our curiosity whether this psychological theory is still functioning for the investment activity. If it does work, we then might establish some investment models for investors to take suitable strategies in advance.

Inspired by the evidence that game rule change might cause behavioral change, we consider that it would be worthwhile to investigate the issue of environment-behavior change in the finance area. We speculate that the regulation change probably results in the change of investor’s behavior in return. On Feb. 1, 2016, Taiwanese government permits to unwind the regulation of day trading in stock market, which makes market participants to believe that the liquidity of market may be improved and the trade of stocks may have more flexibility. Even further, the relaxation may reduce the risk of overnight shareholdings for investors and may protect the interests of investors. However, we argue that there are some issues still unanswered after the relaxation. For example, whether individual and institutional investors, including foreign institutional investors, domestic institutional investors, and security dealers, would prefer trading or holding the stocks with higher day trading ratios, even higher turnover ratios? Does any difference exist for the preference of trading or holding these stocks among individual and institutional investors? These unresolved issues arouse our interest to organize the research.

**P. 6-P. 9**

H1. The shareholding and trading behaviors of investors would be affected by the enhanced day trading volume after the relaxation of day trading regulation.

H2. The shareholding and trading behaviors of individual and institutional investors would be impacted by the relaxation of day trading regulation.

H3. The shareholding and trading behaviors of institutional investors would be affected by the relaxation of day trading regulation.

H4. The margin level and credit trading would be affected by the relaxation of day trading regulation.

H5. The behaviors of individual investors would be related to the issues of corporate governance and financial statement.

4. Literature needs to be updated. It is not clear which literature body your paper may contribute to.

**Response** **to valuable comment:**

Thank you for your precious comment. We agree that we should review the articles cited in the literature to connect with the content of this study more closely. Hence, we adopt several updated papers and drop out unrelated citations. The revised paragraphs are revealed on each subsection of the Literature review and the updated references are displayed from P. 21 to P. 25.

**The following contents are shown in the context of this paper:**

**P. 4- P. 5**

**Intraday trading and day trading**

Intraday traders buy and sell financial instruments in the short term, typically within the same trading day [40]. In other words, intraday trading has an open position that is not left overnight [41] and its observations seem suitable for examining the volume–volatility relation [42]. The intraday data allows us to measure the immediate impact of a certain issue on stock returns and provides a finer picture that cannot be readily extracted from aggregating daily observations. In fact, the advantage of intraday information is particularly valuable if a certain issue influences stock returns more significantly at certain trading hours, for example, at the market opening periods [5].

Day trading, defined as the purchase and sale of the same stock by the same investor on the same day [15], has garnered growing attention in the investment world, especially from investors who are attracted to the prospect of earning high profits by investing only a few hours per day [43]. Although day trading has gained extensive popularity among investors, its profit potential remains a controversial issue [44]. Greater day trading activity leads to greater return volatility and the impact of a day trading shock dissipates gradually within an hour [45]. Besides, day trading increases the bid-ask spread, price depth, and stock volatility, indicating that day trading activities not only cause higher transaction costs and trading risk but also raise the market’s ability to absorb price impact. In sum, the impacts of stock day trading on market quality are not all positive. [46].

**P. 6**

**Institutional and individual investors**

Due to having perverse security selection abilities and limited information, individual investors often sell the winning investments, hold the losing investments, make undiversified stock portfolios, and engage in repeating the past behaviors [52]. In fact, most of the trading losses for individual investors could be traced to their aggressive orders which likely cause the individual investors to suffer losses in stock markets [30]. On average, individual investors lose money from trading [15]. In general, they buy stocks that earn subpar returns and sell stocks that earn strong returns [53]. However, some individual investors might adjust their behavior, which improves their investment performance consequently [54].

Essentially, institutional investors consisted of foreign institutional investors (FIIs), domestic institutional investors (DIIs), and security dealers (SDs), act as vital roles in the business world [55].

**P. 7-P.8**

**Diverse investors and day trading**

Although individual day traders provide market liquidity by reducing the bid‐ask spread, temporary price volatility, and the temporal price impacts, most of them tend to behave as irrational contrarian traders [67]. As a result, individual investor trading results in systematic and economically large losses [30]. Aside from their aggressive orders as mentioned in the previous subsection, a large proportion of individual investors are underdiversified, and the extent of under-diversification is greater for investors who hold only retirement accounts [68]. Particularly, domestic individuals, accounting for the largest portion of total day trading activity, face substantial losses from day trading and individual day traders who trade more frequently and heavily are more likely to suffer such losses. On the contrary, domestic money managers and foreign institutional investors generally make substantial profits through day trading [69].

**P. 21-P. 25**

1. Clitheroe Jr, H. C.; Stokols, D.; Zmuidzinas, M. Conceptualizing the context of environment and behavior. J. Environmental Psychology. 1998, 18(1), 103-112.
2. Moradhaseli, S.; Ataei, P.; Norouzi, A. Analysis of students’ environmental behavior in the Agriculture College of Tarbiat Modares University, Iran (application of Planned Behavior Theory). J. Human Behavior in the Social Environment. 2017, 27(7), 733-742.
3. Gkargkavouzi, A.; Halkos, G.; Matsiori, S. Environmental behavior in a private-sphere context: Integrating theories of planned behavior and value belief norm, self-identity and habit. Resources, Conservation and Recycling. 2019, 148, 145-156.
4. Basri, H.;Amin, S.; Umiyati, M.; Mukhlis, H.; Irviani, R. Learning theory of conditioning. Learning. 2020, 7(8), 2024-2031.
5. Chang, S. C.; Chen, S. S.; Chou, R. K.; Lin, Y. H. Weather and intraday patterns in stock returns and trading activity. J. Banking Financ. 2008, 32(9), 1754-1799.

40.Baralis, E.; Cagliero, L.; Cerquitelli, T.; Garza, P.; Pulvirenti, F. Discovering profitable stocks for intraday trading. Information Sciences. 2017, 405, 91-106.

41.Xin, L.; Lam, K.; Philip, L. H. Effectiveness of filter trading as an intraday trading rule. Stud. Econ. Financ. 2019.

42.Darrat, A. F.; Rahman, S.; Zhong, M. Intraday trading volume and return volatility of the DJIA stocks: A note. J. Banking Financ. 2003, 27(10), 2035-2043.

43.Liew, P. X.; Lim, K. P.; Goh, K. L. Does proprietary day trading provide liquidity at a cost to investors?. Int. Rev. Financ. Anal. 2020, 68, 101455.

44.Kuo, S. H.; Tu, T. T. The Profitability of Day Trading and the Characteristics of Traders: Evidence from the Taiwan Futures Market. Int. Rev. Accounting, Banking Financ. 2020, 12(3), 16.

45.Chung, J. M.; Choe, H.; Kho, B. C. The impact of day‐trading on volatility and liquidity. Asia‐Pacific J. Financ. Stud. 2009, 38(2), 237-275.

46.Yang, T. Y.; Huang, S. Y.; Tsai, W. C.; Weng, P. S. The impacts of day trading activity on market quality: evidence from the policy change on the Taiwan stock market. J. Derivatives Quant. Stud. 2020, 28(4), 191-207.

53.Odean, T. Do investors trade too much?. American Econ. Rev. 1999, 89(5), 1279-1298.

55.Huang, P.; Ni, Y.; Cheng, Y. Does “Resetting” by Changing Corporate Name or Industry Category Appeals to Institutional Investments?. Contemporary Manage. Res. 2019, 15(3), 175-203.

61.	Ferreira, M. A.; Massa, M.; Matos, P. Shareholders at the gate? Institutional investors and cross-border mergers and acquisitions. Rev. Financ. Stud. 2010, 23(2), 601-644.

68.	Goetzmann, W. N.; Kumar, A. Equity portfolio diversification. Rev. Financ. 2008, 12(3), 433-463.

87.	Hsiao, C. Panel data analysis—advantages and challenges. Test. 2007, 16(1), 1-22.

88.	Hsiao, C. Analysis of panel data (No. 54). Cambridge University Press, Cambridge. 2014.

5. There are some issues in the empirics too. Authors think that the endogeneity problem might not be serious in this research. However, the statistic tests for the IV 2SLS and GMM are not presented. And, I do not think using IV 2SLS and GMM as well as Hausman statistics can prove the two independent variables are exogenous.

**Response** **to valuable comment:**

We truly appreciate your constructive comments. Because of turnover ratio affected by firm scales, we then use firm scales as an instrument variable, which might imply that turnover ratio would be exogenous as shown insignificant Huusman tests by employing 2SLS and GMM approaches. Similar results are shown for daytrading ratio as well. In order to save space, we then explain here.

**ivregress gmm foreingtrading daytradingratio (turnoverratio = firmscales) director bigten manager pledge ceoduality boardsize indepdummy** **debtratio assetturnoverratio netprofitratio cashflowratio** **electronicdm, vce(unadjusted)**

**est store IV_reg**

**reg foreingtrading daytradingratio turnoverratio director bigten manager pledge ceoduality boardsize indepdummy debtratio assetturnoverratio netprofitratio cashflowratio** **electronicdm**

**est store LS_reg**

**hausman IV_reg LS_reg**

---- Coefficients ----

| (b) (B) (b-B) sqrt(diag(V_b-V_B))

| IV_reg LS_reg Difference S.E.

-------------+----------------------------------------------------------------

turnoverra~o |	243.1099	-.9550023	244.0649	105.7559

daytrading~o |	-157.6539	-11.54232	-146.1116	142.2766

director |	2.743037	-.1354781	2.878515	1.289202

bigten |	3.147636	-.0101374	3.157773	1.680155

manager |	3.230182	.0077809	3.222401	1.428217

pledge |	-.0246466	-.0547114	.0300648	.2601561

ceoduality |	-29.37603	-2.292998	-27.08303	16.46556

boardsize |	11.77506	1.52628	10.24878	4.654944

indepdummy |	29.94947	-1.56913	31.5186	23.65889

debtratio |	.1479116	.0871041	.0608076	.2656556

assetturno~o |	-22.89333	3.555542	-26.44887	13.86496

netprofitr~o |	-.09701	.0028976	-.0999076	.1328993

cashflowra~o |	-.0131763	.0303554	-.0435316	.1241028

electronicdm |	-28.79385	4.457176	-33.25103	21.47277

------------------------------------------------------------------------------

b = consistent under Ho and Ha; obtained from ivregress

B = inconsistent under Ha, efficient under Ho; obtained from regress

Test: Ho: difference in coefficients not systematic

chi2(15) = (b-B)'[(V_b-V_B)^(-1)](b-B)

=	6.43

**Prob>chi2 =	0.9717**

**ivregress 2sls foreingtrading daytradingratio (turnoverratio = firmscales) director bigten manager pledge ceoduality boardsize indepdummy** **debtratio assetturnoverratio netprofitratio cashflowratio** **electronicdm, first**

**est store IV_reg**

**reg foreingtrading daytradingratio turnoverratio director bigten manager pledge ceoduality boardsize indepdummy debtratio assetturnoverratio netprofitratio cashflowratio electronicdm**

**est store LS_reg**

**hausman IV_reg LS_reg**

---- Coefficients ----

| (b) (B) (b-B) sqrt(diag(V_b-V_B))

| IV_reg LS_reg Difference S.E.

-------------+----------------------------------------------------------------

turnoverra~o |	243.1099	-.9550023	244.0649	122.2036

daytrading~o |	-157.6539	-11.54232	-146.1116	85.25695

director |	2.743037	-.1354781	2.878515	1.476725

bigten |	3.147636	-.0101374	3.157773	1.934468

manager |	3.230182	.0077809	3.222401	1.661387

pledge |	-.0246466	-.0547114	.0300648	.2966378

ceoduality |	-29.37603	-2.292998	-27.08303	17.03619

boardsize |	11.77506	1.52628	10.24878	5.523451

indepdummy |	29.94947	-1.56913	31.5186	22.73928

debtratio |	.1479116	.0871041	.0608076	.2792552

assetturno~o |	-22.89333	3.555542	-26.44887	15.75222

netprofitr~o |	-.09701	.0028976	-.0999076	.1085325

cashflowra~o |	-.0131763	.0303554	-.0435316	.0764813

electronicdm |	-28.79385	4.457176	-33.25103	20.22016

------------------------------------------------------------------------------

b = consistent under Ho and Ha; obtained from ivregress

B = inconsistent under Ha, efficient under Ho; obtained from regress

Test: Ho: difference in coefficients not systematic

chi2(15) = (b-B)'[(V_b-V_B)^(-1)](b-B)

=	3.99

**Prob>chi2 =	0.9978**

6. There should be a separate section for discussions with a focus on how your findings may make theoretical contributions.

**Response** **to valuable comment:**

Thank you for your generous comments. In fact, we discuss the revealed results in the Empirical findings section directly. By doing so, we consider that we might express the denotations and the implications for the real world in time. Nevertheless, we do develop a Discuss section to discuss the models employed in this study for the purpose of robustness. The paragraphs are presented on P. 18 and P. 19.

**The following contents are shown in the context of this paper** **(P. 18 & P. 19):**

Discussion

In general, panel data models should be more appropriate to be used than traditional linear models for employing firm-year observations. Panel data normally submits data containing time series observations of a number of individuals and offers researchers a large number of data points with lowering collinearity among explanatory variables, which enhance the efficiency of econometric estimates. However, as disclosed that the biases in the standard errors vary widely and even are incorrect in many cases, we employ Petersen models in this study for grasping the relative accuracy, which would be beneficial for the robustness of the empirical results.

Nevertheless, for the robustness of the empirical results, we also employ the traditional panel data models (because of employing firm-year observations) and censored panel data models (due to concerning the characteristics of dependent variables) in this study. The results derived by adopting the aforementioned models are similar to those by employing Petersen Models as shown in Tables 3-4. These findings indicate that our revealed results are robust after concerning different models employed.

7. If this paper aims to assess the policy effect of 2016 policy, a PSM-DID model may be a better model to deal with the obvious selection biases.

**Response** **to valuable comment:**

We really appreciate your valuable comment. First, we understood the advantage of analyzing the policy effects using the PSM-DID model, however, we couldn’t compose the entire data due to the data constrained. Considering the stock market after the relaxing policy of day trading is being in a new stage and unreversed trend. We then tried to investigate the behavior relationships in a new stage after the policy relaxing. Secondly, we select the data for the years 2016 and 2017. As the news reported by TWSE, 907 stocks eligible for day trading account for 98.75% of the total market capitalization in TWSE after the relaxation of the day trading regulation. Thus, the selection biases are not mentioned here.

Reviewer #2

1. It is always preferable to provide a paper for review with line numbering. In the absence of line numbering, it is hard to suggest correction exactly where needed.

**Response** **to valuable comment:**

Thank you for your treasured comment. We conduct this study with double-spacing lines. By increasing the space between lines, we expect to have more easy readability for this study.

2. The whole manuscript contains many wordy sentences. Such sentences may be split into small untestable pieces.

**Response to valuable comment:**

We truly appreciate your constructive comments. We agree that several sentences in this study are of long wordings. Therefore, we reedit the whole paper and make sentences easier and more comfortable to be read.

3. Is it mandated by the PLOS ONE author guidelines to abbreviate Literature Review section heading as “Literature Rev.”? if not it may be written fully.

**Response** **to valuable comment:**

Thank you for your constructive comment. As you suggested, we rewrite the section heading of the Literature review and the revision is displayed on P. 4.

**The following contents are shown in the context of this paper** **(P. 4):**

**Literature Review and hypotheses proposed**

4. The intext citations suffer some problems. Several citations are not consistent with the guidelines of PLOS ONE. Citations at the end of sentences are correctly done, while direct citations are not. For example, in literature review section Chan, Chockalingam, and Lai, McInish and Wood, Patell and Wolfson, Stephan and Whaley, and many more throughout the manuscript are not aligned with the citation guidelines. Authors may take care of these issues.

**Response** **to valuable comment:**

Thank you for your valuable comment. As you commented, we have checked and revised the expression of citations for the whole study.

5. Defining abbreviations may be fixed carefully. For example, TWSE is not defined and directly used. However, later, defined in Descriptive statistics section. Similarly, Taiwan Economic Journal (TEJ) is defined twice? Once here and second in Descriptive statistics section.

**Response** **to valuable comment:**

We truly appreciate your constructive comments. We agree with you that we should define the terms used in this study in proper order. Thus, we state the definition of TWSE at the beginning of the Data subsection firstly, as shown on P. 10, and use the abbreviation on the Descriptive statistics section, as presented on P. 12. Simultaneously, we do the similar revision on the expression of TEJ, as displayed on P. 10.

**The following contents are shown in the context of this paper** **(P. 10 & P. 12):**

**P. 10**

Data

We collect daily data of day trading volume from the website of Taiwan Stock Exchange (TWSE) over the period of 2016-2017 due to the concern of data consistency.

(In the second paragraph)

…The former could be measured as mentioned above, and the latter could be derived from Taiwan Economic Journal (TEJ)…

**P. 12**

Descriptive statistics

By utilizing 1,577 observations of TWSE listed firms over the period of 2016-2017 as our samples, Table 2 presents the descriptive statistics, including the number of observations, means, medians, standard deviations, minima, and maxima of the variables employed in this study.

6. Data stationarity condition is one of the basic for regression, wither time series estimator is used or panel. May the authors take into consideration the first- and second-generation panel unit-root and report the results as core Table inside the text and use those results as a predecessor to select the model to be estimated.

**Response** **to valuable comment:**

Thank you for your precious comment. According to the suggestion of the Stata manual, the manual explains that when the number of time periods, T, is small (less than 10 or 15), the test suffers from severe size distortions when fixed effects or time trends are included; in these cases, using altt (make sample adjustment to T) results in much improved size properties at the expense of significantly less power. As a result, we might not take into consideration the first- and second-generation panel unit-root due to employing two year data (i.e. T=2 far less than 10). In addition, differencing time series data such as stock prices and oil prices would be often necessary due to the concern of stationary; however, the movements of the board structure variables (e.g. director holding ratio) and investors holding variables (e.g. foreign holding ratio) in subsequent years might not the same as those of stock prices and oil prices due to stationary concern.

7. Detection and dealing the outliers is initial stage activity, which may not be reported and discuss after estimating VIF. It is surprising to me how 15 regressors are free from multicollinearity. Thus, it is suggested to report VIF, and also Hansen test results as appendices, to help understand readership about the data properties.

**Response** **to valuable comment:**

We truly appreciate your constructive comments. We then report the VIF after detecting and dealing with the outliers at the initial stage. The VIF values for these variables are less than three as shown below, which might indicate that these variables might be free from multicollinearity.

Program for VIF tests:

. reg foreingtrading daytradingratio turnoverratio director bigten manager pledge ceoduality boardsize indepdummy debtratio assetturnoverratio netprofitratio cashflowratio electronicdm firmscales, vce(robust)

. vif

Variable |	VIF	1/VIF

-------------+----------------------

daytrading~o |	2.72	0.367046

turnoverra~o |	2.18	0.459021

electronicdm |	1.79	0.559053

cashflowra~o |	1.52	0.656257

firmscales |	1.47	0.678484

boardsize |	1.39	0.717155

debtratio |	1.37	0.729033

netprofitr~o |	1.36	0.734362

manager |	1.30	0.769412

director |	1.26	0.795768

assetturno~o |	1.16	0.863615

ceoduality |	1.07	0.931624

indepdummy |	1.07	0.933312

bigten |	1.06	0.941964

pledge |	1.04	0.961842

-------------+----------------------

Mean VIF |	1.45

8. P12, authors stated that “……owing to the defects of the panel data models proposed by Petersen [72], we, therefore, employ the model proposed by Petersen for grasping the relative accuracy after taking the structure of the data into account.”. May the mentioned defect be highlighted, and how these can mislead the estimation? Moreover, it is expected to provide detailed advantages of the Petersen model opted by the authors. What other related methods are available in the field and how the selected model is superior over all of them?

**Response** **to valuable comment:**

Thank you for your generous comments. As you suggested, we address the drawback of panel data models in the Discussion section. To avoid the widely varying biases in the standard errors generated by panel data models, we employ Petersen models in this study and expect to enhance the robustness of the empirical results. The expression is presented on P. 18 and P. 19.

**The following contents are shown in the context of this paper (P. 18 & P. 19):**

**Discussion**

In general, panel data models should be more appropriate to be used than traditional linear models for employing firm-year observations. Panel data normally submits data containing time series observations of a number of individuals and offers researchers a large number of data points with lowering collinearity among explanatory variables, which enhance the efficiency of econometric estimates. However, as disclosed that the biases in the standard errors vary widely and even are incorrect in many cases, we employ Petersen models in this study for grasping the relative accuracy, which would be beneficial for the robustness of the empirical results.

9. Petersen (2008) have outlined comprehensively on how to cluster the standard error in various cases. Therefore, the authors are expected to articulate the selection of model taking guide from Petersen (2008).

**Response** **to valuable comment:**

We really appreciate your valuable comment. As mentioned in the previous comment, we employ Petersen models in this study to avoid the widely varying biases in the standard errors generated by panel data models. The description is revealed on P. 18 and P. 19.

**The following contents are shown in the context of this paper** **(P. 18 & P. 19):**

**Discussion**

In general, panel data models should be more appropriate to be used than traditional linear models for employing firm-year observations. Panel data normally submits data containing time series observations of a number of individuals and offers researchers a large number of data points with lowering collinearity among explanatory variables, which enhance the efficiency of econometric estimates. However, as disclosed that the biases in the standard errors vary widely and even are incorrect in many cases, we employ Petersen models in this study for grasping the relative accuracy, which would be beneficial for the robustness of the empirical results.

10. I would be interested to know how Petersen (2008) deals with cross-sectional dependence in used panel dataset. By the way, have authors tested cross-sectional dependence? If yes, what were the results?

**Response** **to valuable comment:**

Thank you for your treasured comment. In this study, instead of concerning cluster between year and firm (i.e. Petersen models), the model concerning the clustered between firms (i.e. concerning cross-sectional dependence) is suggested by Petersen (2008). We then reveal that the results are similar to those with concerning clustered between firm and year. We infer that similar findings might result from the results adjusted among clustered residuals only. In order to save space, we explain here.

Program for the concern of cross-sectional dependence by concerning clustered between firms (i.e. adding robust cluster(firm) at the end of the following model as shown below.

**Stata Programming Instructions for Petersen website: https://www.kellogg.northwestern.edu/faculty/petersen/htm/papers/se/se_programming.htm**

**Clustered (Rogers) Standard Errors – One dimension**

**regress *dependent_variable independent_variables*, robust cluster(*cluster_variable*)**

**regress foreingtrading daytradingratio turnoverratio director bigten manager pledge ceoduality boardsize indepdummy debtratio assetturnoverratio netprofitratio cashflowratio electronicdm firmscales, robust cluster(firm)**

**Clustered Standard Errors – Two dimensions**

**cluster2 *dependent_variable independent_variables*, cluster(*cluster_variable_one*)  tcluster(*cluster_variable_two*)**

11. Inconsistent decimal places in Table 2 and subsequent Tables may be streamlined. It is standard in our field to use up to three decimal places. The word table may be written as “Table” intext where it is referred.

**Response** **to valuable comment:**

We truly appreciate your constructive comments. Concerning the consistent decimal places in Table 2, the subsequent Tables should be streamlined (i.e. up to three decimal places). However, due to 0.000 might be sometimes appeared if employing up to three decimal places only, we suggest that our empirical results might be properly presented up to five decimal places in Tables 3-4. Besides, the word table would be written as “Table” where it is referred to in this paper. Thank you for your suggestion for the consistency of the paper presentation. We are really appreciated it.

12. I guess line spacing is not aligned with rest of the document, and Petersen, 2009 departs from PLOS ONE reference style.

**Response** **to valuable comment:**

Thank you for your constructive comment. As you mentioned, we have checked and revised the expression of citations for the whole study.

13. May the authors explain the reason behind relatively high constant in 1A, 3A? why it is equally low in the other two models?

**Response** **to valuable comment:**

We really appreciate your treasured comment.

**The value and range dependent variable as presented in Table 2**

14. Discussion section preferably be separated as independent section before the conclusion section.

**Response** **to valuable comment:**

Thank you for your valuable comment. As you suggested, we develop a Discuss section to discuss the models employed in this study for the purpose of robustness. The paragraphs are presented on P. 18 and P. 19.

**The following contents are shown in the context of this paper** **(P. 18 & P. 19):**

Discussion

In general, panel data models should be more appropriate to be used than traditional linear models for employing firm-year observations. Panel data normally submits data containing time series observations of a number of individuals and offers researchers a large number of data points with lowering collinearity among explanatory variables, which enhance the efficiency of econometric estimates. However, as disclosed that the biases in the standard errors vary widely and even are incorrect in many cases, we employ Petersen models in this study for grasping the relative accuracy, which would be beneficial for the robustness of the empirical results.

Nevertheless, for the robustness of the empirical results, we also employ the traditional panel data models (because of employing firm-year observations) and censored panel data models (due to concerning the characteristics of dependent variables) in this study. The results derived by adopting the aforementioned models are similar to those by employing Petersen Models as shown in Tables 3-4. These findings indicate that our revealed results are robust after concerning different models employed.

15. The research touches an interesting area, while results may be supported by performing some robustness (an alternative measure of dependent/independent variable, alternative estimator, out of sample analysis or reduced sample analysis, or maybe with additional control). It is suggested to create a section before discussion as Robustness. The authors do have the liberty to perform at least any two robustness out of few suggested.

**Response** **to valuable comment:**

We truly appreciate your constructive comments. we develop a Discuss section to discuss the models employed in this study for the purpose of robustness. The paragraphs are shown on P. 18 and P. 19.

**The following contents are shown in the context of this paper** **(P. 18 & P. 19):**

Discussion

In general, panel data models should be more appropriate to be used than traditional linear models for employing firm-year observations. Panel data normally submits data containing time series observations of a number of individuals and offers researchers a large number of data points with lowering collinearity among explanatory variables, which enhance the efficiency of econometric estimates. However, as disclosed that the biases in the standard errors vary widely and even are incorrect in many cases, we employ Petersen models in this study for grasping the relative accuracy, which would be beneficial for the robustness of the empirical results.

Nevertheless, for the robustness of the empirical results, we also employ the traditional panel data models (because of employing firm-year observations) and censored panel data models (due to concerning the characteristics of dependent variables) in this study. The results derived by adopting the aforementioned models are similar to those by employing Petersen Models as shown in Tables 3-4. These findings indicate that our revealed results are robust after concerning different models employed.

16. The sub-heading “Research Implications and further studies” may be omitted”. It is sufficient to discuss implications and future directions as plain text.

**Response** **to valuable comment:**

Thank you for your precious comment. We adopt your suggestion and delete the sub-heading “Research Implications and further studies.” The revised paragraphs are displayed on P. 20 and P. 21.

**The following contents are shown in the context of this paper** **(P. 20 & P. 21):**

By revealing that the shareholding and trading behaviors would be different among diverse investors, we claim that these revealed results might provide several implications that enrich the existing literature. First, we illustrate that investors might observe and even trace the behaviors of successful investors, such as FIIs, to enhance their investment performance, especially after the relaxation of day trading regulation in Taiwan. Furthermore, aside from focusing on the effects of day trading or turnover ratio on the shareholding and trading behaviors of diverse investors, we might have more proxies, such as accelerating turnover ratio except day trading ratio proposed in this study, to conduct more researches.

Second, in addition to the relaxation of day trading regulation, we argue that corporate governance, financial performance, and firm scale should be concerned because FIIs prefer holding or trading the stocks probably based on these issues.

As with all research, this study has some limitations, which provides the direction for future research. First, by utilizing the data of TWSE listed firms due to the limited availability of data resources, the revealed results of this study might be likely twisted. Since the scales of TWSE listed firms are somewhat small, the values of these firms might be affected by the short-term large capital inflows and outflows. Second, as an emerging market of TWSE, the empirical results of this study might be different from those of developed markets. For future research, we should have more representative markets and find other crucial variables to conduct further studies related to affect the shareholding and trading of diverse investors.

17. The first sentence of conclusion consists of almost 5 lines, which would be confusing to the readers. May authors carefully split such wordy sentence into small untestable sentences throughout the manuscript.

**Response** **to valuable comment:**

We truly appreciate your constructive comments. We agree with you that the first sentence of the Conclusion is too wordy. Therefore, we reorganize this paragraph and separate the expression into three short sentences. The revised text is shown on P. 19.

**The following contents are shown in the context of this paper** **(P. 19):**

At the beginning of 2016, the day trading of stocks is expanded considerably in Taiwan, which arouses our interest to investigate whether the shareholding and trading volume of diverse investors would be affected by day trading ratio and turnover ratio. Therefore, we organize this research to explore these issues by incorporating several variables, such as board structure, financial statements, and other as controlling variable. This study includes FIIs, DIIs, SDs, and individual investors as diverse investors and reveals several impressive findings in return.

18. Few lines may be added on limitations of the current piece of research; then future directions will be adequate.

**Response** **to valuable comment:**

Thank you for your generous comments. We adopt your suggestion and address the limitation and further research for this study. The paragraph is shown on P. 21.

**The following contents are shown in the context of this paper** **(P. 21):**

As with all research, this study has some limitations, which provides the direction for future research. First, by utilizing the data of TWSE listed firms due to the limited availability of data resources, the revealed results of this study might be likely twisted. Since the scales of TWSE listed firms are somewhat small, the values of these firms might be affected by the short-term large capital inflows and outflows. Second, as an emerging market of TWSE, the empirical results of this study might be different from those of developed markets. For future research, we should have more representative markets and find other crucial variables to conduct further studies related to affect the shareholding and trading of diverse investors.

Reviewer #3

1. The motivation needs to be strengthened.

**Response** **to valuable comment:**

We really appreciate your valuable comment. We agree with you that we should strengthen the motivation of this study because motivation is the original intention to conduct research. If the motivation is strong enough, researchers might generate high quality work that attracts people to cite. Hence, we reorganize the motivation paragraph in the Introduction section and attempt to improve the value of this study. The revision is displayed on P. 3.

**The following contents are shown in the context of this paper** **(P. 3):**

We conduct this study to examine whether the relaxation of day trading regulation would change the diverse investors' behaviors, which is stemmed from the following motivations. First, we attempt to identify if the speculative individual investors might prefer investing stocks with higher day trading ratios and, in contrast, if the other individual deemed as the uninformative traders might not prefer trading these stocks due to the difficulty of making profits. Second, as realized with making more effort on fundamentals instead of speculation, institutional investors should tend to decrease the shareholdings and trading volume of stocks with higher day trading ratios. However, some institutional investors would prefer trading and holding the stocks with higher day trading ratios, especially for those having unreleased information. Therefore, we endeavor to solve the puzzle of whether these institutional investors have this preference due to liquidity concerns.

2. The main estimated coefficients need to be interpreted.

**Response** **to valuable comment:**

Thank you for your treasured comment. We would endeavor to interpret the main estimated coefficients in this study. For example, Table 3 reveals that day trading ratio and turnover ratio negatively relate to the foreign shareholding ratio (β =-0.0189299**, P< 0.05; β =-0.0035782, P<0.05) but positively relate to the market level ratio (β =0.0999011***, P<0.01; β =-0.0388799, P<0.01). This result indicates that FIIs might not prefer holding the stocks with higher day trading ratio and turnover ratio but individual investors prefer holding these stocks.

3. It may be useful to test for the presence of significant heteroscedasticity.

**Response** **to valuable comment:**

We truly appreciate your constructive comments.

After running the models without concerning heteroscedasticity issues, we then reveal that heteroscedasticity issues would be serious in our models as shown Breusch-Pagan / Cook-Weisberg test for heteroskedasticity as shown below

**. estat hettest**

**Breusch-Pagan / Cook-Weisberg test for heteroskedasticity**

**Ho: Constant variance**

**Variables: fitted values of foreingtrading**

**chi2(1)	=	412.37**

**Prob > chi2 =	0.00001**

As a result, due to significant heteroscedasticity existed in our models, we then use the comment vce(robust) recommend by Stata to deal with heteroscedasticity issues in our models.

4. To establish robustness, it may be useful to also apply an alternative estimation technique.

**Response** **to valuable comment:**

Thank you for your constructive comment. In fact, instead of employing Petersen regression models, we also employ traditional panel data models and even censored panel data models for robustness. The results are similar to those revealed in this study. Because of concerning shareholding and trading behaviors of diverse investors, the length of this paper would be regarded as a long paper. We then explain the above concern in footnote 12.

5. The wiring needs improvement.

**Response** **to valuable comment:**

Thank you for your generous comments. We have amended this study completely. We hope that this study is expressed clearer and easier to read after the modification.
