# Supplementary material for: Are the shareholding and trading behaviors of diverse investors affected by the relaxation of day trading?
Source: PLoS One. 2021 Apr 23;16(4):e0250121. doi: 10.1371/journal.pone.0250121 (PMC8064584; doi:10.1371/journal.pone.0250121)
Supplement: Attachment — Submitted filename: Response_to_Reviewers_0327.docx [file pone.0250121.s003.docx]

Reviewers' comments:

Reviewer #2

1. The point was to insert line numbering, while response is double-spacing which has nothing to do with line numbering. Line numbering helps referring exact point where authors may make correction. There are 5 authors in this paper and none of them understand the difference between line numbering and line spacing. Go to Layout menu, click on and chose. I hope it will be understandable now to 5 authors.

Thank you for your precious comment. We are so sorry for missing the line numbering on the previous revision. As you suggested, we insert the numbers of lines for the whole paper and hope to improve the readability. Again, we truly appreciate your warm reminding.

2. There is no point to use diplomatic wording iteratively that “We truly appreciate your constructive comments” and do nothing to address the comment. In Table 2 if 0 may appear, so 0.000 may also appear which has same meanings. There are many numbers in 2 decimals, few in 3 others in none. Suppose we accept your argument of 5 decimal place, why same is not done in Table 2.?

**Response** **to valuable comment:**

We deeply appreciate your treasured comment. We are so sorry for missing to modify the numbers in Table 2 on the previous revision. As you suggested, we revise the numbers in Table 2 in 5 decimals, except the observations. The modification is shown on P. 13 to P. 14. Once more, thanks for your kind reminding.

**The following contents are shown in the context of this paper** **(P. 13 to P. 14):**

**Table 2 Descriptive statistics**

| Variables | Obs. | Mean | Median | SD | Max | Min |
| --- | --- | --- | --- | --- | --- | --- |
| Foreign shareholding ratio (%) | 1577 | 13.96100 | 8.59000 | 15.66700 | 89.21000 | 0.01000 |
| Institutional shareholding ratio (%) | 1577 | 0.48000 | 0.00000 | 1.38900 | 20.21000 | 0.00000 |
| Dealer shareholding ratio (%) | 1577 | 0.11000 | 0.01000 | 0.58300 | 10.00000 | 0.00000 |
| Margin level ratio (%) | 1577 | 11.79900 | 7.67000 | 12.97400 | 98.81000 | 0.00000 |
| Foreign trading ratio (%) | 1577 | 14.04000 | 9.07000 | 15.13700 | 84.20000 | 0.00000 |
| Institutional trading ratio (%) | 1577 | 0.69400 | 0.00000 | 2.85800 | 32.14000 | 0.00000 |
| Dealer trading ratio (%) | 1577 | 1.42300 | 0.05000 | 2.98000 | 24.69000 | 0.00000 |
| Credit trading ratio (%) | 1577 | 1.16000 | 0.23000 | 2.69700 | 34.31000 | 0.00000 |
| Day trading ratio | 1577 | 15.62500 | 10.31000 | 15.70000 | 103.08800 | 0.04400 |
| Turnover ratio | 1577 | 122.59600 | 64.89800 | 168.79400 | 2404.45500 | 0.61340 |
| Debt ratio | 1577 | 41.78300 | 41.88000 | 17.67400 | 96.54000 | 0.90000 |
| Asset turnover ratio | 1577 | 0.79800 | 0.70000 | 0.56600 | 5.35000 | 0.01000 |
| Net profit ratio (%) | 1577 | 4.07500 | 5.49000 | 57.84100 | 465.83000 | -1613.22000 |
| Cash flow ratio (%) | 1577 | 28.40800 | 21.69000 | 72.32400 | 719.74000 | -1463.88000 |
| Directors’ holding ratio (%) | 1577 | 21.86900 | 17.93000 | 15.40000 | 94.55000 | 0.01000 |
| Top 10 shareholding ratio (%) | 1577 | 1.51100 | 0.27000 | 4.55300 | 77.83000 | 0.00000 |
| Managers' holding ratio (%) | 1577 | 24.00400 | 21.72000 | 12.78700 | 73.25000 | 0.51000 |
| Directors’ pledge ratio (%) | 1577 | 8.05400 | 0.00000 | 15.55400 | 97.69000 | 0.00000 |
| .CEO duality | 1577 | 0.29900 | 0.00000 | 0.45800 | 1.00000 | 0.00000 |
| Board size | 1577 | 7.96000 | 7.00000 | 2.26800 | 20.00000 | 2.00000 |
| Independent director dummy | 1577 | 0.93900 | 1.00000 | 0.23900 | 1.00000 | 0.00000 |
| Electronic dummy | 1577 | 0.46300 | 0.00000 | 0.49800 | 1.00000 | 0.00000 |
| Firm scales | 1577 | 15.79700 | 15.60200 | 1.32800 | 22.50600 | 12.80800 |

3. Comment 13. It was asked to explain the reason behind relatively high constant in 1A, 4A? why it is equally low in the other two models? Which is responded in nonsense why that “The value and range dependent variable as presented in Table 2”. By the way what descriptive statistics has to do with justification of constant reported in other Tables?

**Response** **to valuable comment:**

According to the regression model, the constant is often defined as the [mean](https://statisticsbyjim.com/glossary/mean/) of the [dependent variable](https://statisticsbyjim.com/glossary/response-variables/) when we set all of the [independent variables](https://statisticsbyjim.com/glossary/predictor-variables/) in the model to zero. Thus, the dependent variable with high mean might have a relatively high constant. Besides, if the positive (negative) relationship exists between the dependent variable and most of the independent variables, the constant term is likely to be positive (negative) in the model.

As a result, the means of foreign shareholding ratio and margin level ratio are higher than those of domestic institutional shareholding ratio and security dealer shareholding ratio, which is the reason to that the constants of 1A and 4A are higher than those of 2A and 3A. We then learn that descriptive statistics shown in Table 2 would be related to the constants reported in Tables 3. Besides, similar findings are shown in Table 4. Thank you so much for your valuable comment.

**The following contents are shown in the context of this paper** **(P. 13 & P. 15):**

**Table 2 Descriptive statistics**

| Variables | Obs. | Mean | Median | SD | Max | Min |
| --- | --- | --- | --- | --- | --- | --- |
| Foreign shareholding ratio (%) | 1577 | 13.96100 | 8.59000 | 15.66700 | 89.21000 | 0.01000 |
| Institutional shareholding ratio (%) | 1577 | 0.48000 | 0.00000 | 1.38900 | 20.21000 | 0.00000 |
| Dealer shareholding ratio (%) | 1577 | 0.11000 | 0.01000 | 0.58300 | 10.00000 | 0.00000 |
| Margin level ratio (%) | 1577 | 11.79900 | 7.67000 | 12.97400 | 98.81000 | 0.00000 |

**Table 3 Results of shareholding behaviors for diverse investors**

| Dependent variable | (1A)  Foreign  Institutional  Shareholding  ratio | | (2A)  Domestic  Institutional shareholding ratio | | (3A)  Security  Dealer  shareholding ratio | | (4A)  Margin  level  ratio | |
| --- | --- | --- | --- | --- | --- | --- | --- | --- |
| Independent variable |  |  |  |  |  |  |  |  |
| Constant | | -101.8849*** | | -2.88669*** | | 0.16446 | | 50.50962*** |
|  | | (4.385597) | | (0.27228) | | (0.11804) | | (4.66163) |

4. The paper may be professionally copyedited.

**Response** **to valuable comment:**

Thank you for your valuable comment. We have someone who is good in the English writing academically to reedit this paper. We hope the paper being easier to be read after the modification.
